# Myosin 1b Regulates Nuclear AKT Activation by Preventing Localization of PTEN in the Nucleus

**DOI:** 10.1016/j.isci.2019.07.010

**Published:** 2019-07-11

**Authors:** Yi Yu, Yuyan Xiong, Diogo Ladeiras, Zhihong Yang, Xiu-Fen Ming

**Affiliations:** 1 Cardiovascular and Aging Research, Department of Endocrinology, Metabolism and Cardiovascular System, Medicine Section, Faculty of Science and Medicine, University of Fribourg, Chemin du Musée 5, 1700 Fribourg, Switzerland

**Keywords:** Biochemistry, Biological Sciences, Cell Biology, Molecular Biology

## Abstract

Insulin-induced AKT activation is dependent on phosphoinositide 3-kinase and opposed by tumor suppressor phosphatase and tensin homolog (PTEN). Our previous study demonstrates that myosin 1b (MYO1B) mediates arginase-II-induced activation of mechanistic target of rapamycin complex 1 that is regulated by AKT. However, the role of MYO1B in AKT activation is unknown. Here we show that silencing MYO1B in mouse embryonic fibroblasts (MEF) inhibits insulin-induced nuclear but not cytoplasmic AKT activation accompanied by elevated nuclear PTEN level. Co-immunoprecipitation, co-immunostaining, and proximity ligation assay show an interaction of MYO1B and PTEN resulting in reduced nuclear PTEN. Moreover, the elevated nuclear PTEN upon silencing MYO1B promotes apoptosis of MEFs and melanoma B16F10 cells. Taken together, we demonstrate that MYO1B, by interacting with PTEN, prevents nuclear localization of PTEN contributing to nuclear AKT activation and suppression of cell apoptosis. This may present a therapeutic approach for cancer treatment such as melanoma.

## Highlights

• MYO1B, by interacting with PTEN, prevents PTEN localization in the nucleus
• MYO1B prevents nuclear localization of PTEN depending on its motor activity
• This contributes to nuclear AKT activation and suppression of cell apoptosis
• Targeting MYO1B may represent a therapeutic approach for cancer treatment

## Graphical Abstract

**Figure:**
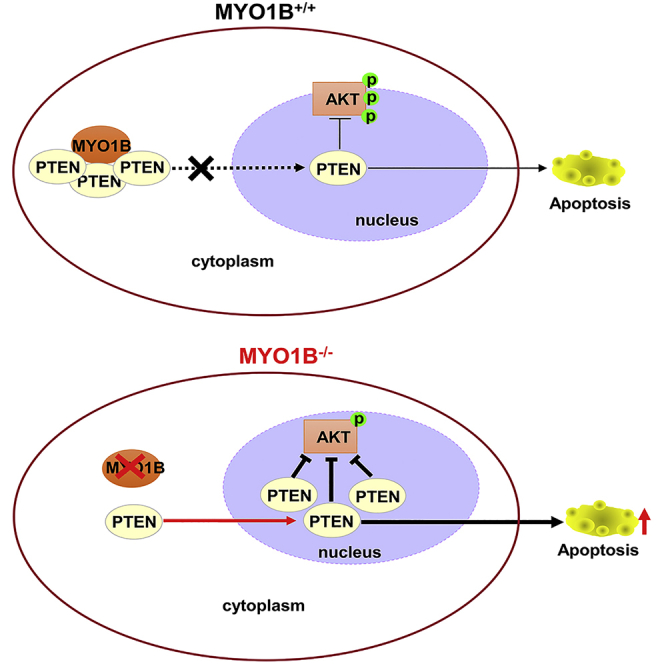

## Introduction

AKT, also known as protein kinase B (PKB), is a serine/threonine kinase that plays a crucial role in a variety of cellular processes including cell growth, proliferation, and metabolism (Mackenzie and Elliott, 2014, Nguyen et al., 2006). AKT is activated by various stimuli such as insulin in a phosphoinositide 3-kinase (PI3K)-dependent manner. Phosphatidylinositol-3,4,5-trisphosphate (PI(3,4,5)P3) produced by PI3K recruits AKT to the plasma membrane via binding to the pleckstrin homology (PH) domain of AKT, which enables phosphorylation of AKT at Thr308 within the T-loop of the catalytic domain by PI3K-dependent kinase 1 (PDK1), and at Ser473 within the carboxyl-terminal hydrophobic domain by mechanistic target of rapamycin complex 2 (MTORC2) (Burgering and Coffer, 1995, Cheng et al., 2005, Franke et al., 1995, Gonzalez and McGraw, 2009, Mora et al., 2004). Hyperactive AKT has been found in a variety of tumors (Altomare and Testa, 2005, Yoeli-Lerner and Toker, 2006).

Currently, the majority of investigations focused on cytosolic AKT. Studies, however, also demonstrate the presence of active AKT in the nucleus (Borgatti et al., 2003, Leinninger et al., 2004, Meier et al., 1997, Mistafa et al., 2008). It has been reported that nuclear AKT is involved in cell cycle progression, cell survival, DNA repair, RNA export, cell differentiation, and tumorigenesis (Martelli et al., 2012). Only little information is currently available with regard to the mechanisms underlying the regulation of nuclear AKT activation. It has been reported that in PC12 cells, cytoplasmic AKT phosphorylation accompanied by nuclear AKT phosphorylation and translocation upon nerve growth factor stimulation was modulated by PI3K activity (Nguyen et al., 2006). It has also been reported that cytoplasmic AKT was able to translocate to the nucleus without phosphorylation as demonstrated in HEK293 cells, indicating that phosphorylation of AKT was not required for its nuclear localization (Saji et al., 2005). Although it remains elusive how AKT is activated within the nucleus (Ananthanarayanan et al., 2005, Andjelkovic et al., 1997, Liu and Brown, 2011), activated nuclear AKT is reported in various cancers such as lung, breast, and prostate cancers as well as in acute myeloid leukemia (Cappellini et al., 2003, Lee et al., 2002, Nicholson et al., 2003, Van de Sande et al., 2005). Hyperactive AKT in nucleus has been also observed in invasive head and neck carcinoma cell lines and in glioblastomas (Giudice et al., 2011, Suzuki et al., 2010). Moreover, a study showed that long-term treatment of statins exhibited anticancer effects in A549 lung cancer cells, which is accompanied by a decline in nuclear AKT-Thr308 levels (Miraglia et al., 2012). All these studies suggest an important role of hyperactive nuclear AKT in carcinogenesis. Evidences have been presented that hyperactive nuclear AKT is involved in tumorigenesis by promoting proliferation and maintenance of stemness in cancer stem cells by phosphorylation and inactivation of the cell cycle inhibitory protein CDKN1A (Jain et al., 2015), as well as by its antiapoptotic effect (Lee et al., 2008, Rubio et al., 2009).

The PI3K-AKT signaling pathway is antagonized by the tumor suppressor, phosphatase and tensin homolog (PTEN), which catalyzes the conversion of phosphatidylinositol-3,4,5-triphosphate (PI(3,4,5)P3) to phosphatidylinositol-4,5-biphosphate (PI(4,5)P2) in the cytoplasm (Cantley and Neel, 1999, Downes et al., 2007, Liu et al., 2009). The PTEN signaling pathway is implicated in the regulation of cell metabolism, growth, proliferation, survival, and migration, and its aberration causes tumorigenesis (Bassi et al., 2013). Mutations of PTEN are frequently detected in a variety of human cancers (Li et al., 1997, Parsons, 2004). PTEN was originally identified as a cytoplasmic protein; subsequently, multiple studies show that both cytoplasmic and nuclear PTEN exist and both exhibit tumor suppressive function (Milella et al., 2015, Planchon et al., 2008). Solid evidence indicates that PTEN is localized primarily in the nucleus of normal quiescent cells, whereas neoplastic cells possess dominantly cytoplasmic PTEN, suggesting that it is the nuclear PTEN that exerts the tumor suppressor function (Planchon et al., 2008, Whiteman et al., 2002). The cytoplasmic PTEN exerts tumor suppressive effect mainly by antagonizing the PI3K-AKT-dependent cell growth and survival, whereas the nuclear PTEN suppresses tumor by multiple mechanisms independent of PI3K-AKT, including stabilizing another tumor suppressor TP53 by interacting with p53, inhibiting cyclin D1 expression, inducing the expression of RAD51 and thus enhancing DNA repair, and promoting ubiquitin-dependent degradation of oncoproteins such as Polo-like kinase 1 and aurora kinase AURK (Chen et al., 2018, Milella et al., 2015). It is to be noted that nuclear PTEN is capable of impairing carcinogenesis by promoting cell apoptosis. Accumulation of nuclear PTEN in U87MG human glioblastoma cells upon treatment with apoptotic stimuli, such as tumor necrosis factor-α or doxorubicin, indicates a proapoptotic role for the nuclear PTEN (Gil et al., 2006).

Myosin 1b (MYO1B) is an actin-binding motor protein that is categorized as the monomeric, nonfilamentous class-1 myosin (Komaba and Coluccio, 2010). Studies on localization and subcellular fractionation suggest that MYO1B associates with the plasma membrane and certain subcellular organelles such as endosomes and lysosomes (Komaba and Coluccio, 2010). Our most recent study demonstrates that MYO1B serves as a mediator in arginase-II (ARG2)-induced activation of the MTORC1 that is regulated by AKT (Yu et al., 2018). However, the role of MYO1B in insulin-induced AKT activation has not been investigated. In this study, by knocking down MYO1B in immortalized mouse embryonic fibroblasts (MEFs) and hepatocyte AML12 cells, we demonstrate that MYO1B, by interacting with PTEN, prevents localization of PTEN in the nucleus, favoring nuclear AKT activation and cell survival in various cell types including melanoma cells. This finding reveals a regulatory mechanism of nuclear PTEN-AKT pathway linked to cell apoptosis including in melanoma cells.

## Results

### MYO1B Is Required for Insulin-Induced AKT Activation

To explore a role of MYO1B in insulin-induced AKT activation, the effect of knocking down MYO1B on AKT activation was examined. As shown in Figure S1, silencing MYO1B significantly suppressed short-term insulin-induced AKT activation (15-min stimulation) as monitored by AKT phosphorylation at Thr308 and Ser473 in immortalized MEFs (Figures S1A and S1B) and hepatocytes (Figures S1C and S1D). It is to be noted that silencing MYO1B in both cells did not influence the insulin-induced activation of MTORC1-RPS6K1 pathway as monitored by ribosomal protein RPS6 phosphorylation at Ser240/244 (Figure S1), indicating that MYO1B is not required for insulin-induced MTORC1-RPS6K1 activation. To determine which AKT isoform is activated, AKT-T308 and AKT-S473 of AKT1, AKT2, and AKT3 were analyzed by immunoblotting after immunoprecipitation using the isoform-specific AKT antibody. As shown in Figure S2, all the three AKT isoforms are activated upon stimulation and modulated by MYO1B. The most significant effect of MYO1B silencing was on the AKT2 isoform. Moreover, silencing MYO1B also significantly attenuated AKT activation upon long-term insulin stimulation in MEFs (2- to 24-h stimulation) (Figure S3).

### MYO1B Plays a Role in Insulin-Induced Nuclear, but Not Cytoplasmic, AKT Activation

**Figure 1:**
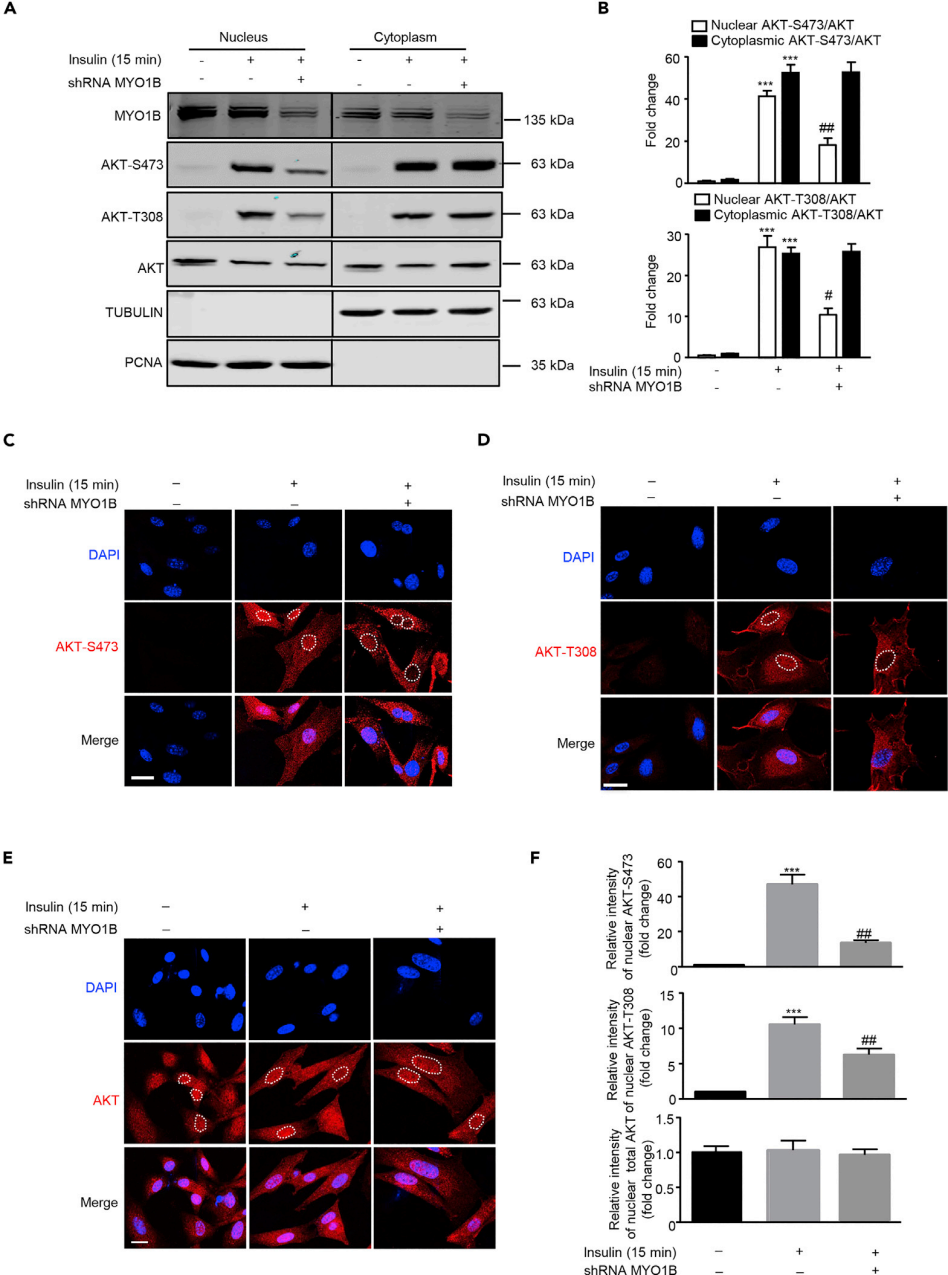
MYO1B Is Required for Insulin-Induced Nuclear AKT Activation Immortalized MEF cells were transduced with rAd/U6-LacZ-short hairpin RNA (shRNA) as control and rAd/U6-MYO1B-shRNA for silencing MYO1B. After 3 days of transduction and 16 h of serum starvation, the cells were treated with 100 nmol/L insulin for 15 min or not treated. (A) Immunoblotting analysis of subcellular distribution of MYO1B and AKT phosphorylation in the nucleus and cytoplasm. TUBULIN and PCNA were used as markers for cytoplasm and nucleus, respectively. The line between the third and fourth lane indicates cutting of the same blots. (B) Quantification of the signals in (A). (C and D) (C) Immunofluorescence staining for AKT-S473 (red) and (D) AKT-T308 (red) were followed by counterstaining with DAPI (blue). The merged images are also shown. White dashes in images outline the area of nucleus. (E) Immunofluorescence staining for AKT total. (F) Quantification of signals of the nuclear AKT-S473 shown in (C), AKT-T308 shown in (D), and AKT shown in (E). Scale bar, 25 μm. All values are presented as mean ± SEM of the data from three independent sets of experiments. One-way ANOVA; ***p < 0.001 versus control; #p < 0.05 and ##p < 0.01 versus insulin. rAd, recombinant adenovirus.

Accumulated evidences highlighted that the nuclear AKT serves as a key component in a variety of signaling pathways (Martelli et al., 2012), which prompted us to explore whether subcellular AKT activation is regulated by MYO1B in MEFs. Immunoblotting analysis of subcellular fractions revealed that silencing MYO1B in MEFs significantly attenuated insulin-induced nuclear AKT activation, but had no effect on cytoplasmic AKT activation (Figures 1A and 1B). This finding was confirmed by confocal microscopic immunostaining as shown in Figures 1C, 1D, and 1F. As the white dotted lines drawn along the nuclear envelope for signal quantification in Figures 1C and 1D may interfere with observation of pAKT near nuclear envelope, images without the white dotted lines are supplied (Figure S4). These images showed no obvious pAKT enrichment near the nuclear envelope. The enhanced or reduced pAKT upon stimulation or silencing MYO1B, respectively, was observed only in the nucleus, but not in the cytoplasm. Of note, total AKT expression levels in nucleus and cytoplasm were not altered upon silencing MYO1B (Figures 1E and 1F). The purity of both nuclear and cytosolic fractions was verified by detection of proliferating cell nuclear antigen (PCNA) (marker of nucleus) and TUBULIN (marker of cytoplasm). These results demonstrate that MYO1B is required for insulin-induced nuclear, but not cytoplasmic, AKT activation.

### MYO1B Regulates Nuclear AKT Activation by Control of Nuclear PTEN Level

**Figure 2:**
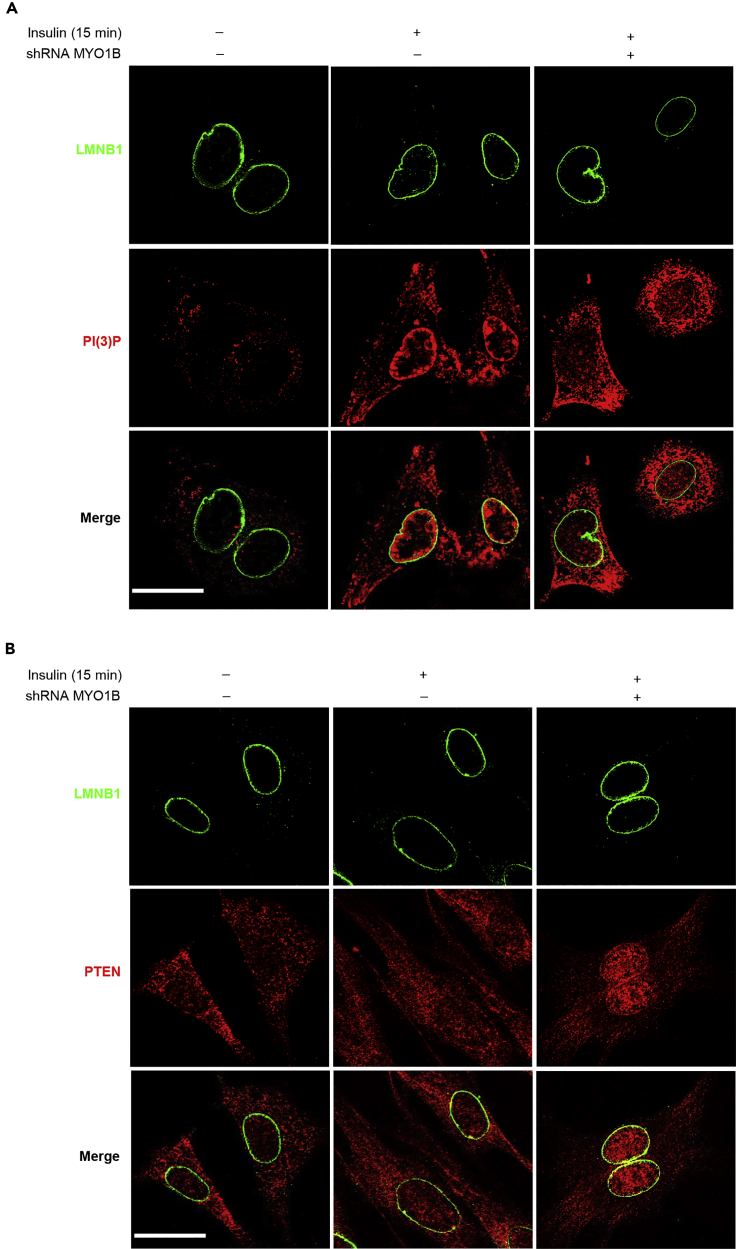
MYO1B Mediates Nuclear PI(3)P and PTEN Distributions (A and B) Immortalized MEF cells were transduced with rAd/U6-LacZ-shRNA as control and rAd/U6-MYO1B-shRNA for silencing. After 3 days of transduction and 16 h of serum starvation, the cells were treated with 100 nmol/L insulin for 15 min or not treated. Co-immunofluorescence staining for LMNB1 (green) and PI(3)P (red) (A) and LMNB1 (green) and PTEN (red) (B). LMNB1 was used as the marker of nuclear envelope. The merged images are also shown. Scale bar, 25 μm. Shown are the representative images from three independent sets of experiments. shRNA, short hairpin RNA.

**Figure 3:**
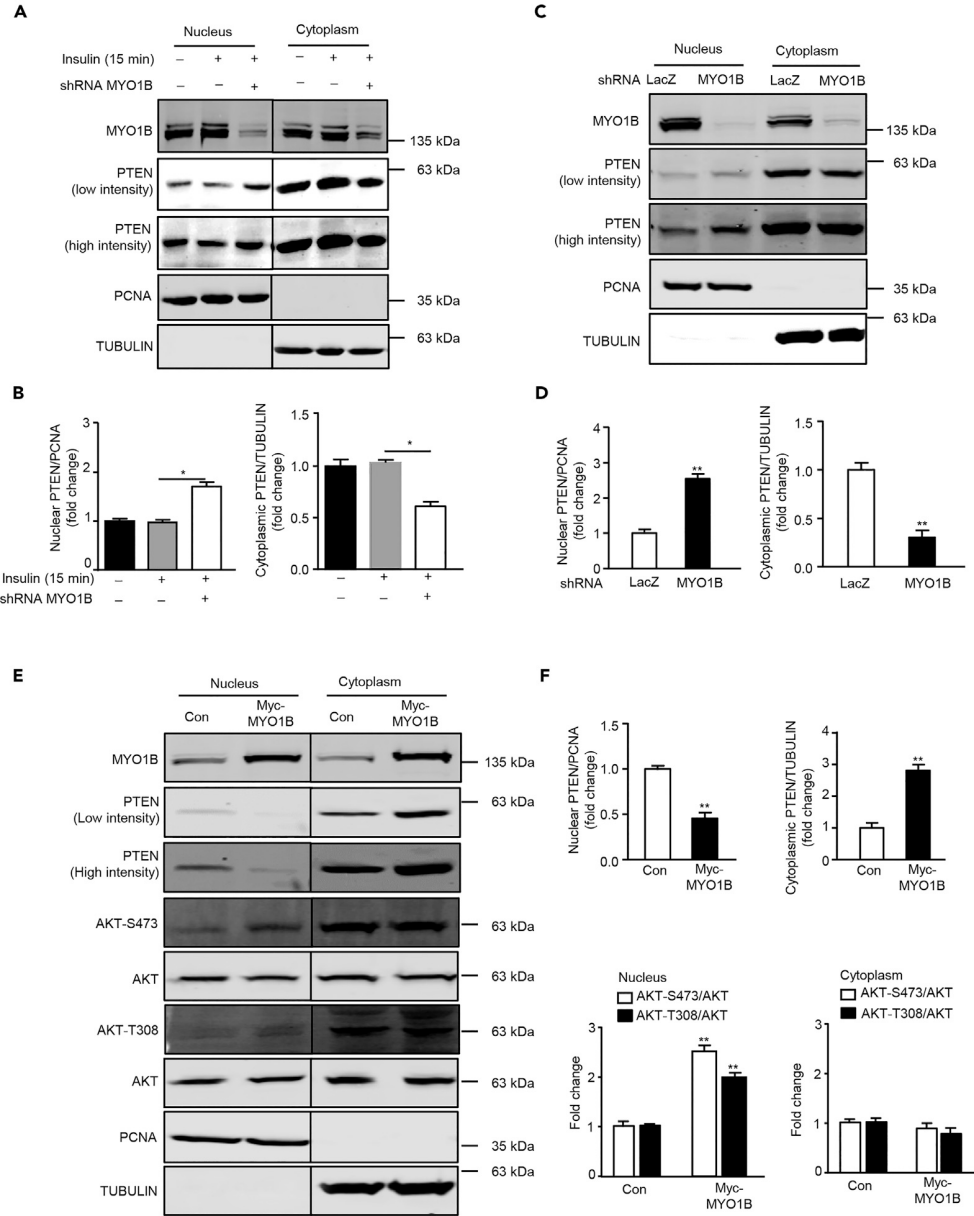
MYO1B Regulates Cytoplasmic and Nuclear PTEN Distribution (A) Experiments were performed as described for Figure 1A. Immunoblotting analysis of nuclear and cytoplasmic fractions was shown. (B) Quantification of the signals in (A). (C) Immortalized MEF cells were transduced as described for Figure 1A, except that cells were not treated with insulin. Immunoblotting analysis of nuclear and cytoplasmic fractions was shown. (D) Quantification of the signals in (C). (E) Immortalized MEF cells were transduced with rAd/CMV-LacZ as control and rAd/CMV-MYO1B for overexpression. After 2 days of transduction and 16 h of serum starvation, immunoblotting analysis of subcellular distribution of PTEN in the nucleus and cytoplasm. (F) Quantification of the signals in (E). TUBULIN and PCNA were used as markers of the cytoplasm and nucleus, respectively. The line between the third and fourth lanes indicates cutting of the same blots. All values are presented as mean ± SEM of the data from three independent sets of experiments. One-way ANOVA (B) or t test (D and F); *p < 0.05 and **p < 0.01 versus control.

The fact that there is no change in total nuclear AKT upon stimulation suggests that the rapid increase in nuclear AKT phosphorylation upon stimulation is unlikely to result from the translocation of activated AKT from the cytoplasm into the nucleus and that the AKT activation mechanisms exist in the nucleus. To confirm this, the subcellular changes of the phosphatidylinositol 3-phosphate (PI(3)P) reflecting PI3K and PTEN activities upon stimulation in the absence or presence of MYO1B^shRNA^ were examined. Co-immunofluorescence staining of PI(3)P and PTEN with a nuclear membrane marker LMNB1 was performed to determine whether there are any PI(3)P and PTEN in the nuclear membrane and their changes upon insulin stimulation and MYO1B knockdown, respectively. Figure 2 reveals that both PI(3)P and PTEN are present in the nucleus. PI(3)P is enriched near the nuclear membrane (Figure 2A), whereas PTEN is distributed throughout the nucleus (Figure 2B). Moreover, nuclear PI(3)P was strongly enhanced in parallel with the increase in cytoplasmic PI(3)P upon insulin stimulation. However, only the enhanced nuclear PI(3)P, particularly that near the nuclear envelope, but not cytoplasmic PI(3)P, was prevented by silencing MYO1B (Figure 2A). Figure 2B shows that PTEN was detected in both the cytoplasm and nucleus. Although the levels of both cytoplasmic and nuclear PTEN did not change upon stimulation, silencing MYO1B markedly enhanced intranuclear and nuclear membrane PTEN levels with simultaneous decrease in cytoplasmic PTEN (Figure 2B). To further explore the potential mechanism by which MYO1B regulates nuclear AKT activation in response to insulin, the level of PTEN was examined in the subcellular fractions of MEFs (Verrastro et al., 2016). In agreement with the results of immunostaining, immunoblotting analysis of subcellular fractions revealed that although the levels of both cytoplasmic and nuclear PTEN did not change upon stimulation (Figures 3A and 3B), silencing MYO1B increased nuclear PTEN with a concomitant decrease in cytoplasmic PTEN either in the presence (Figures 3A and 3B) or absence of insulin treatment (Figures 3C and 3D). In agreement with these results, a decrease in nuclear PTEN with a concomitant increase in cytoplasmic PTEN was observed in MEFs overexpressing MYO1B, which was accompanied by enhanced nuclear AKT activation (Figures 3E and 3F). It is to note that cytoplasmic AKT activation was not affected. These results suggest a role of MYO1B in the regulation of nuclear AKT activation by control of nuclear PTEN level.

### MYO1B Prevents PTEN Nuclear Localization by Binding to PTEN

**Figure 4:**
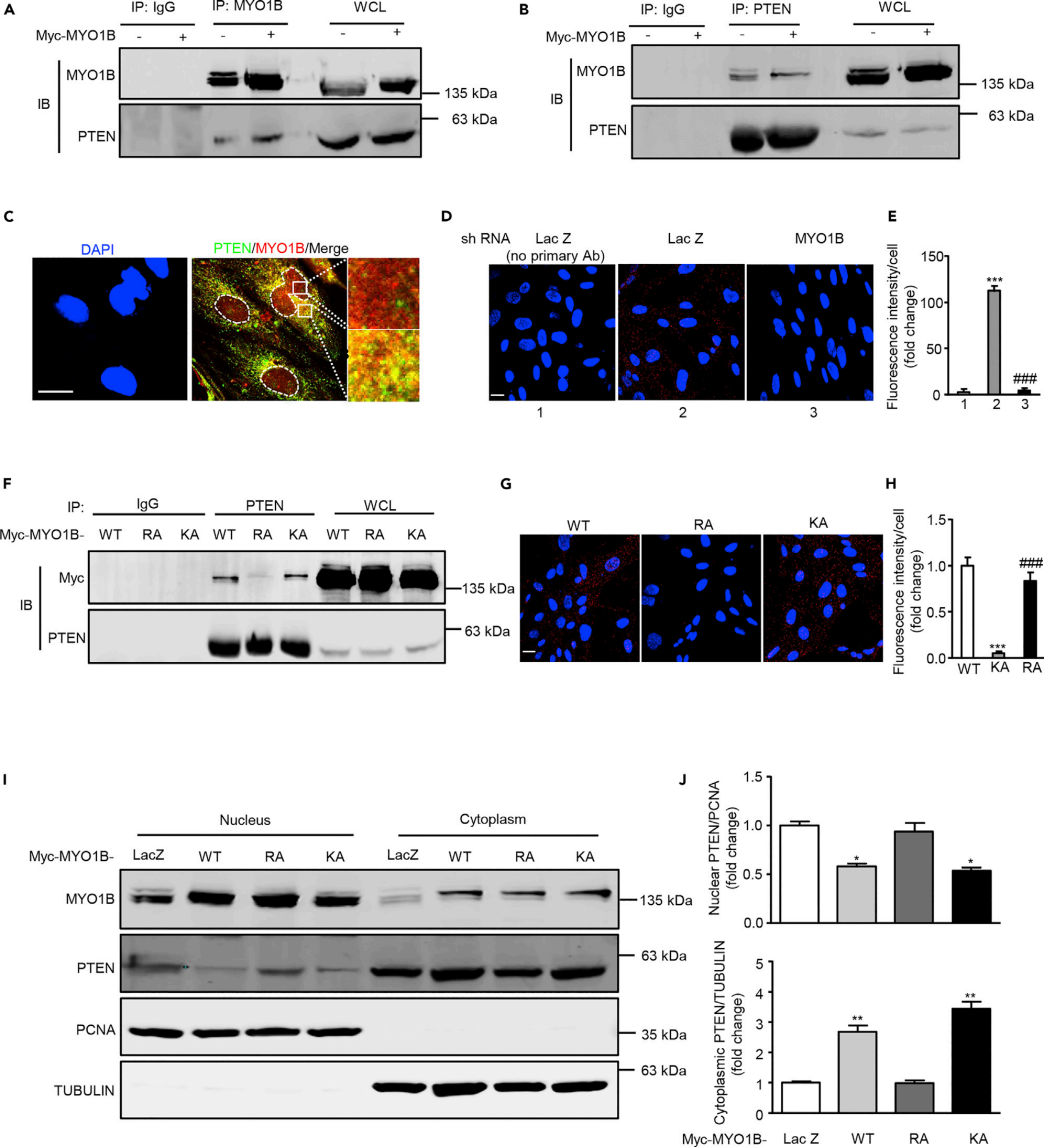
MYO1B interacts with PTEN (A and B) Immortalized MEF cells were transduced with rAd/CMV-LacZ as control and rAd/CMV-MYO1B for overexpression. After 2 days of transduction and 16 h of serum starvation, immunoblotting analysis of MYO1B and PTEN in the whole-cell lysates (WCL) and immunoprecipitates using an anti-MYO1B (A) or PTEN antibody (B) was performed. Immunoprecipitation using a normal IgG served as negative control. (C) Immunofluorescence staining for PTEN (green) and MYO1B (red) was followed by counterstaining with DAPI (blue). The merged images are also shown. The rightmost images are the enlargements of the selected area in the corresponding pictures immediately to the left. White dashes in images outline the area of nucleus. (D) Duolink proximity ligation assay (PLA) for protein interaction between PTEN and MYO1B in MEF cells. Immortalized MEF cells were transduced with rAd/U6-LacZ-shRNA as control and rAd/U6-MYO1B-shRNA for silencing MYO1B. After 3 days of transduction, the Duolink assay was performed as described (cells without primary antibody as the negative control). Each red spot represents a single interaction, and DNA was stained with DAPI. (E) Quantification of the signals in (D). (F) Immortalized MEF cells were transduced with rAd/CMV-myc-MYO1B-wild type (WT), rAd/CMV-myc-MYO1B-R165A (RA), and rAd/CMV-myc-MYO1B-K966A (KA) for overexpression. After 2 days of transduction and 16 h of serum starvation, immunoblotting analysis of Myc-MYO1B and PTEN in the whole-cell lysates (WCL) and immunoprecipitates using an anti-PTEN antibody was performed. Immunoprecipitation using a normal IgG served as negative control. (G) Duolink PLA for protein interaction between PTEN and Myc in MEF cells with the overexpression of MYO1B-WT, MYO1B-RA, and MYO1B-KA. (H) Quantification of the signals in (G). (I) Immortalized MEF cells were transduced with rAd/CMV-LacZ as control, rAd/CMV-myc-MYO1B-WT (WT), rAd/CMV-myc-MYO1B-R165A (RA), and rAd/CMV-myc-MYO1B-K966A (KA) for overexpression. Immunoblotting analysis of subcellular distribution of PTEN in the nucleus and cytoplasm was shown. TUBULIN and PCNA were used as markers of cytoplasm and nucleus, respectively. (J) Quantification of the signals in (I). shRNA, short hairpin RNA. Scale bar, 25 μm. All values are presented as mean ± SEM of the data from three to four independent sets of experiments. One-way ANOVA; *p < 0.05, **p < 0.01, ***p < 0.001 versus control or WT. ###p < 0.001 versus LacZ or KA.

Previous study indicates that MYO1B might also act as a dynamic scaffold in interacting directly or indirectly with other proteins to modulate the trafficking of these proteins (Salas-Cortes et al., 2005). To determine whether MYO1B interacts with PTEN to regulate PTEN nuclear localization, co-immunoprecipitation experiments were performed. As shown in Figure 4A, PTEN was detected in immunoprecipitates of both endogenous and overexpressed MYO1B. Conversely, MYO1B was also co-immunoprecipitated by anti-PTEN antibody (Figure 4B). Moreover, co-immunostaining reveals the co-localization of PTEN and MYO1B, which is mainly observed in the cytoplasm (Figure 4C). The interaction between MYO1B and PTEN is further verified by proximity ligation assay (PLA) (Figures 4D and 4E). To determine which domain of MYO1B is responsible for the interaction of MYO1B and PTEN, two MYO1B mutants, R165A (a mutant in the motor domain) and K966A (a mutant in its C-terminal PH domain), were constructed to examine the effect of these mutations on the interaction of MYO1B and PTEN. Co-immunoprecipitation experiment shows that R165A mutant exhibited markedly reduced capability for interacting with PTEN (Figure 4F), suggesting that R165 within the motor domain plays a crucial role in the interaction of MYO1B with PTEN. These results were also further verified by PLA (Figures 4G and 4H). In accordance, overexpressed R165A mutant failed to prevent the PTEN localization in the nucleus as native or K966 mutant did (Figures 4I and 4J). It is noteworthy that the interaction of MYO1B with PTEN is independent of insulin stimulation (Figure S5). These results demonstrate that MYO1B prevents PTEN nuclear localization by binding to PTEN, which is dependent on the motor domain of MYO1B.

### MYO1B Suppresses Cell Apoptosis through Prevention of PTEN Nuclear Localization

**Figure 5:**
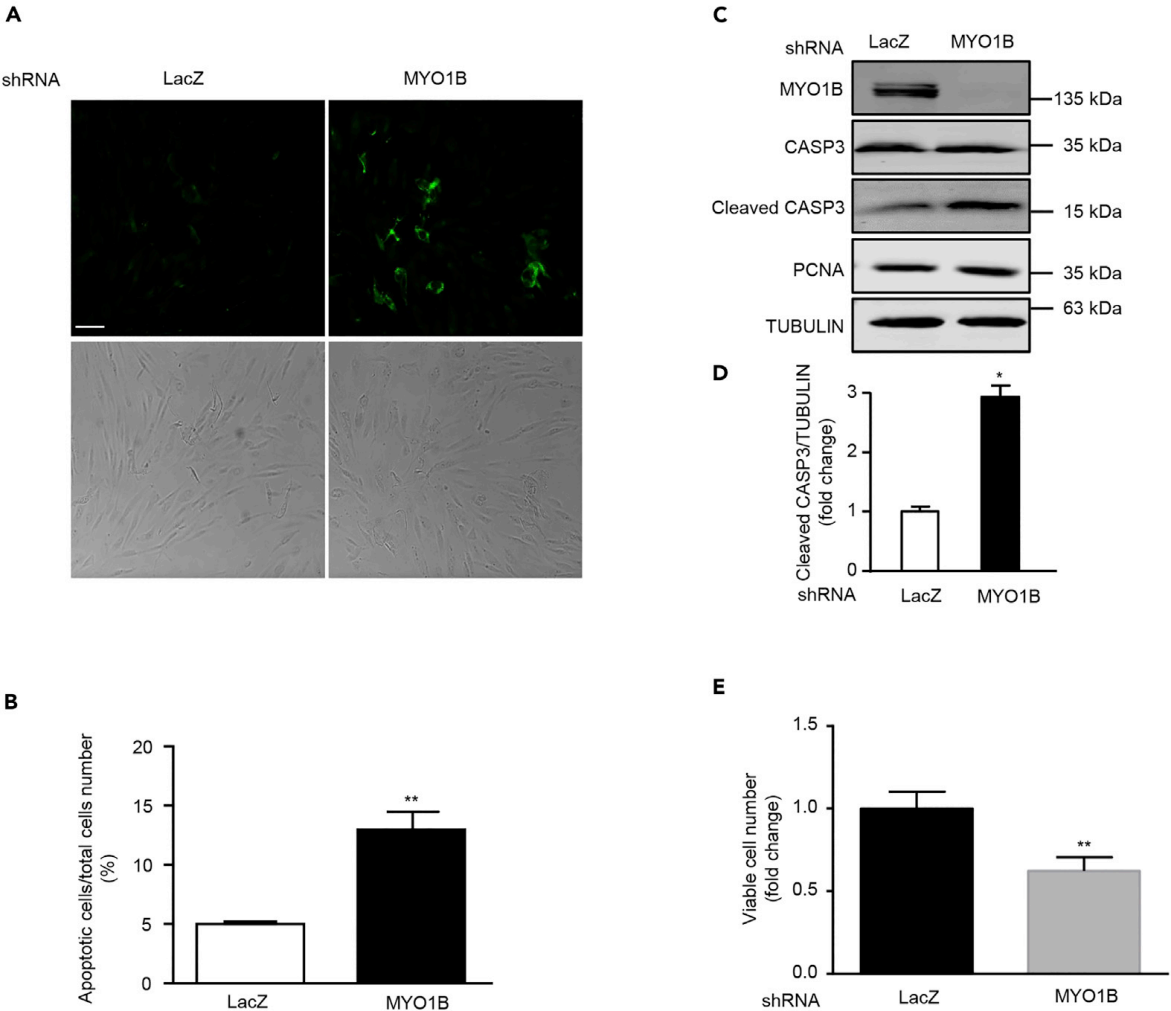
MYO1B Depletion Promotes Apoptosis of Immortalized MEF Cells (A) Immortalized MEF cells were transduced with rAd/U6-LacZ-shRNA as control and rAd/U6-MYO1B-shRNA for silencing MYO1B. After 3 days of transduction and 16 h of serum starvation (A) apoptotic cells were detected by Annexin-V-FLUOS staining (upper panel). The lower panels show phase contrast images. (B) Plot graphs present quantification of Annexin-V-positive apoptotic cells shown in (A). (C) Immunoblotting analysis of MYO1B, caspase 3 (CASP3), cleaved CASP3, and PCNA. (D) Quantification of the signals in (C). (E) Viable cell number. Scale bar, 100 μm. All values are presented as mean ± SEM of the data from three to four independent sets of experiments; t test; *p < 0.05, **p < 0.01 versus LacZ-shRNA control. shRNA, short hairpin RNA.

**Figure 6:**
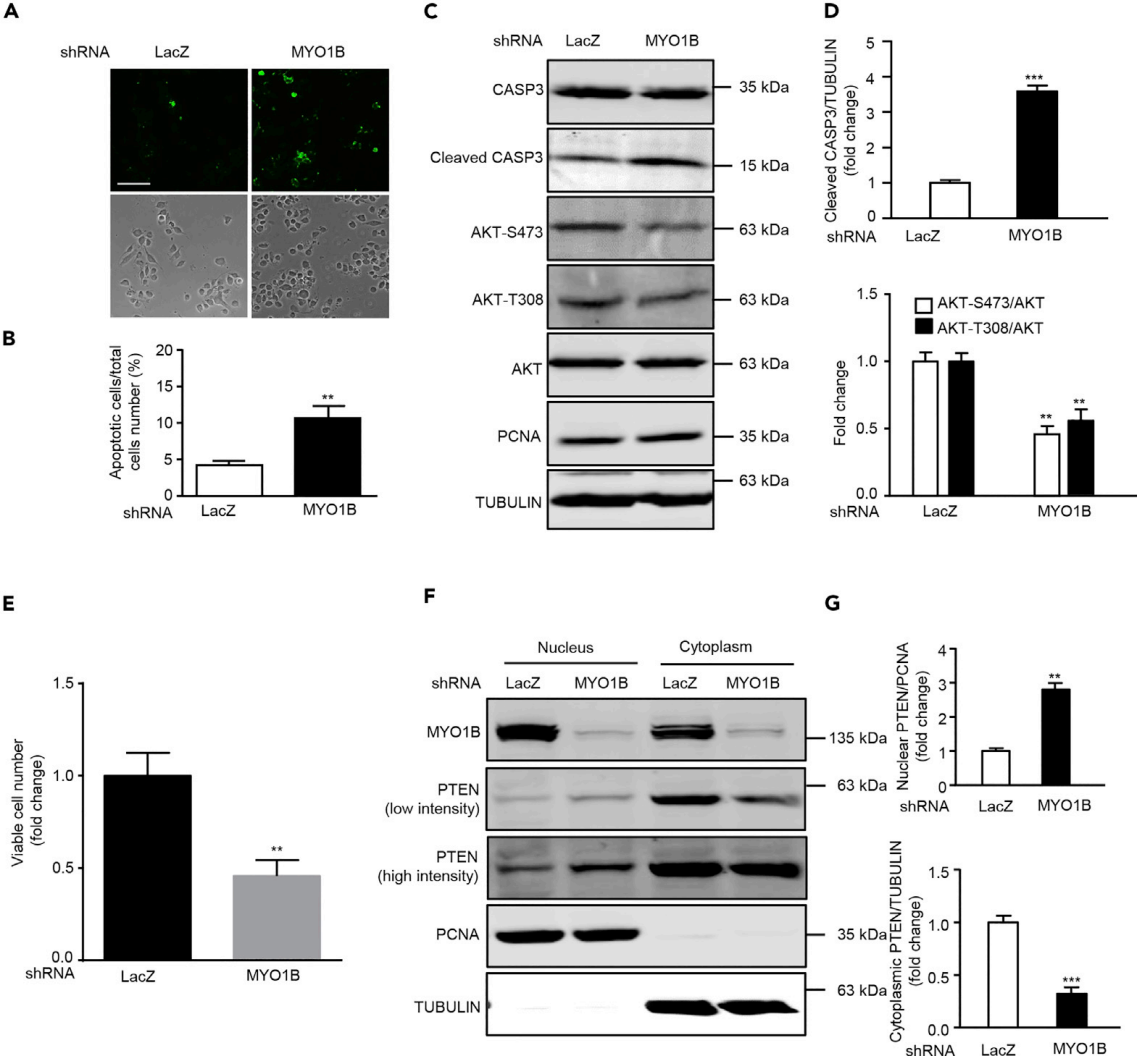
MYO1B Depletion Promotes Apoptosis of Melanoma Cells Experiments were performed as described in Figure 4, except that melanoma B16F10 cells were used. (A) Detection of apoptotic cells by Annexin-V-FLUOS staining (upper panel). The lower panels show phase contrast images. (B) Plot graphs present quantification of apoptotic cells shown in (A). (C) Immunoblotting analysis of caspase 3 (CASP3), cleaved CASP3, AKT-S473, and AKT-T308. (D) Quantification of the signals in (C). (E) Viable cell number. (F) Immunoblotting analysis of subcellular distribution of PTEN in nucleus and cytoplasm. (G) Quantification of the signals in (F). TUBULIN and PCNA were used as marker of the cytoplasm and nucleus, respectively. Scale bar, 100 μm. All values are presented as mean ± SEM of the data from three independent sets of experiments; t test; **p < 0.01, ***p < 0.001 versus LacZ-shRNA control. shRNA, short hairpin RNA.

Emerging evidences demonstrate that nuclear PTEN promotes cell apoptosis (Gil et al., 2006, Planchon et al., 2008). We next wish to investigate the effects of MYO1B-mediated prevention of PTEN nuclear localization on cell apoptosis in immortalized MEFs and melanoma cells. In support of previous reports, silencing MYO1B, which augmented nuclear PTEN level, induced cell apoptosis of MEFs and melanoma cell B16F10 as reflected by enhanced Annexin-V immunostaining (Figures 5A, 5B, 6A, and 6B) and increased cleaved caspase 3 level without a change in PCNA level (Figures 5C, 5D, 6C, and 6D). Moreover, cell survival assay revealed that silencing MYO1B significantly reduced viable survival cell number (Figures 5E and 6E). Consistent with results obtained in MEFs as shown in Figure 3C, silencing MYO1B increased nuclear PTEN with a concomitant decrease in cytoplasmic PTEN in melanoma cells (Figures 6F and 6G). These results suggest that MYO1B suppresses cell apoptosis by prevention of PTEN nuclear localization.

**Figure 7:**
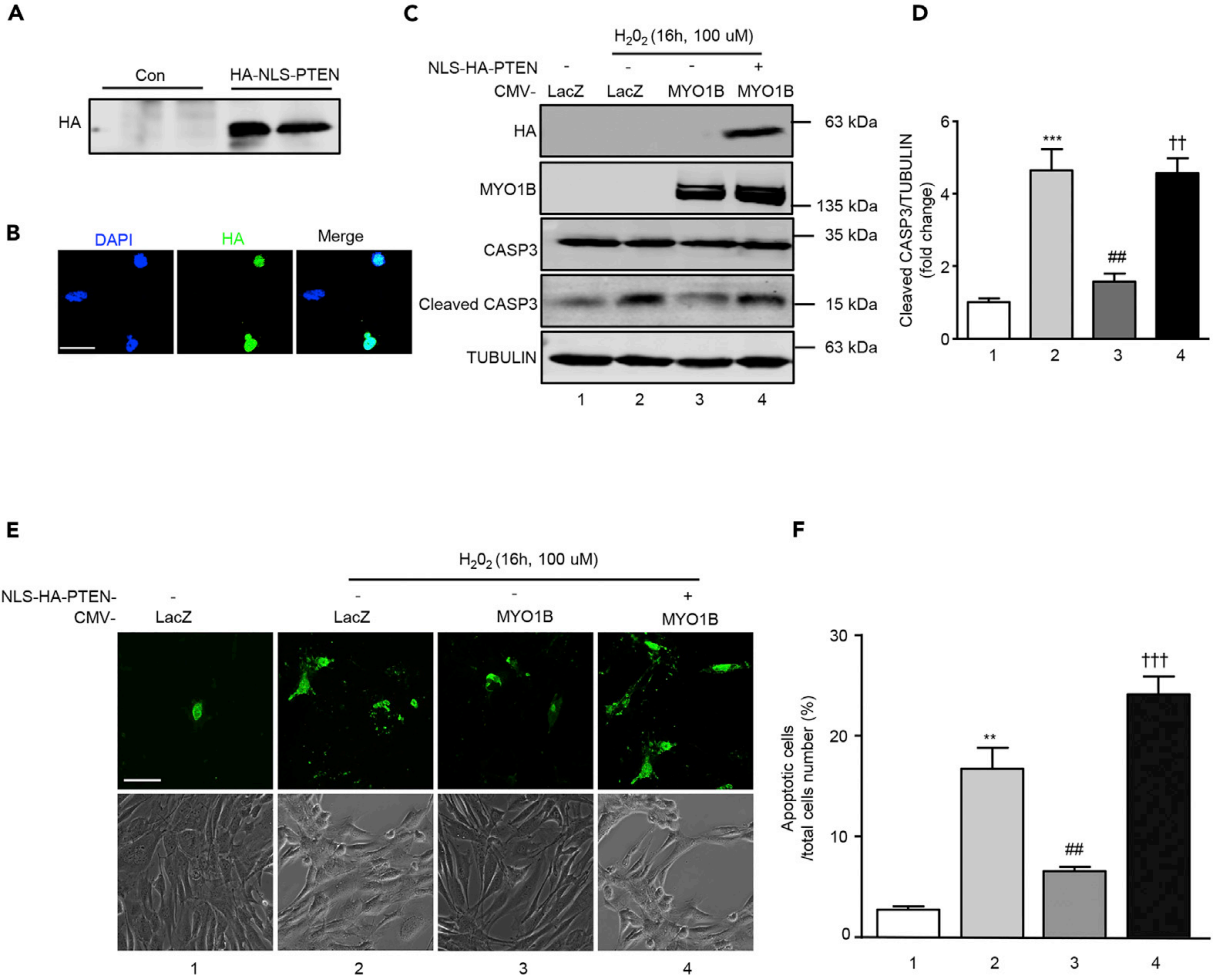
MYO1B Suppresses Cell Apoptosis by Prevention of PTEN Nuclear Localization in MEF Cells (A and B) Immortalized MEF cells were transduced with rAd/CMV-NLS-PTEN for overexpression. After 2 days transduction, immunoblotting analysis (A), and immunofluorescence staining (B) of HA-NLS-PTEN were performed. Scale bar, 25 μm. (C and E) Immortalized MEF cells were transduced with rAd/CMV-LacZ as control and rAd/CMV-MYO1B and rAd/CMV-NLS-PTEN for overexpression. After 2 days of transduction and 16 h of serum starvation, cells were treated with or without 100 μmol/L hydrogen peroxide for 16 h. Immunoblotting analysis for CASP3 and cleaved CASP3 (C) and detection of apoptotic cells by Annexin-V-FLUOS staining (E) were then performed. Scale bar, 100 μm. (D and F) Quantification of the signals in (C) and (E), respectively. All values are presented as mean ± SEM of the data from three independent sets of experiments. One-way ANOVA; **p < 0.01, ***p < 0.001 versus control; ##p < 0.01 versus H_2_O_2_; ††p < 0.01, †††p < 0.001 versus H_2_O_2_ + CMV-MYO1B.

**Figure 8:**
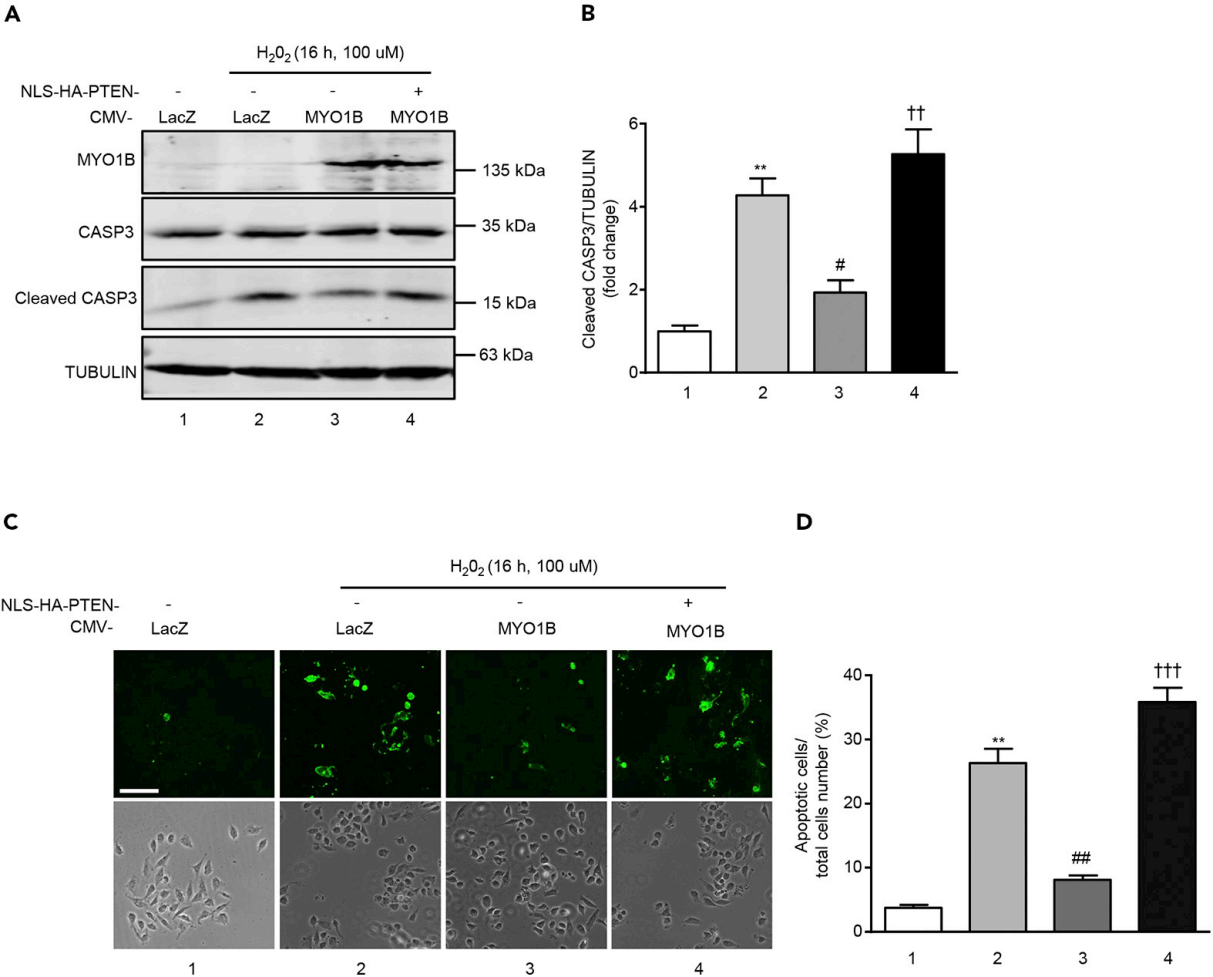
MYO1B Suppresses Cell Apoptosis by Prevention of PTEN Nuclear Localization in Melanoma Cells Experiments were performed as described in Figure 7, except that melanoma B16F10 cells were used. (A) Immunoblotting analysis for CASP3 and cleaved CASP3. (B) Quantification of the signals in (A). (C) Detection of apoptotic cells by Annexin-V-FLUOS staining. (D) Quantification of the signals in (C). Scale bar, 100 μm. All values are presented as mean ± SEM of the data from three independent sets of experiments. One-way ANOVA; **p < 0.01 versus control; #p < 0.05, ##p < 0.01 versus H_2_O_2_; ††p < 0.01, †††p < 0.001 versus H_2_O_2_ + CMV-MYO1B.

To further confirm that MYO1B regulates cell apoptosis by modulating nuclear PTEN level by interacting with PTEN, a hemagglutinin (HA)-tagged PTEN mutant harboring a nuclear localization sequence (NLS) (HA-NLS-PTEN) was constructed. This mutant was exclusively localized in the nucleus (Figures 7A and 7B). Hydrogen peroxide (H_2_O_2_) was used as the stimulus for inducing cell apoptosis owing to the low basic level of cell apoptosis in immortalized MEFs and melanoma cell B16F10. Overexpression of MYO1B suppressed H_2_O_2_-induced MEF cell apoptosis as evaluated by increased cleaved caspase 3 (Figures 7C and 7D) and Annexin-V staining (Figures 7E and 7F). Remarkably, co-overexpression of HA-NLS-PTEN with MYO1B significantly reversed the cell apoptosis suppression caused by overexpressing MYO1B upon H_2_O_2_ treatment (Figures 7C–7F). Consistent results were also obtained in melanoma B16F10 cells (Figure 8). These results provide evidence that MYO1B suppresses cell apoptosis by interacting with PTEN leading to the prevention of PTEN nuclear localization.

## Discussion

Insulin-induced activation of the cytosolic AKT has been well demonstrated (Hagan et al., 2008, Sarbassov et al., 2005, Wick et al., 2000), whereas little is known about the mechanisms underlying the regulation of nuclear AKT activation. In the present study, we identified MYO1B as a regulator of nuclear AKT activation. By knocking down or overexpressing MYO1B in immortalized MEFs or melanoma cells, we demonstrate that MYO1B, by interacting with PTEN, through the motor domain, prevents localization of PTEN in the nucleus, contributing to nuclear AKT activation and suppression of cell apoptosis (see graphical abstract). This represents a regulatory mechanism of nuclear PTEN-AKT pathway linked to cell apoptosis and may present a therapeutic approach for cancer treatment, such as melanoma.

Our recent study shows that MYO1B mediates the effect of ARG2 in activating MTORC1-RPS6K1 through promotion of peripheral lysosomal positioning, which is involved in hyperactive MTORC1-RPS6K1 linking to senescence-associated cell apoptosis in vascular smooth muscle cells (Yu et al., 2018). In the current study, we further investigated a role of MYO1B in insulin-induced activation of MTORC1-RPS6K1 and MTORC2-AKT using immortalized MEFs. The results show that silencing MYO1B does not affect insulin-induced MTORC1-RPS6K1 activation, but attenuates AKT activation by insulin. We thus focused on further elucidation of the role of MYO1B in AKT activation in MEFs. Both immunoblotting analysis of subcellular fractionation and immunofluorescence staining reveal that only nuclear but not cytoplasmic AKT activation is suppressed by silencing MYO1B, indicating that MYO1B plays a critical role in nuclear AKT activation. This conclusion is also supported by experiments of overexpressing MYO1B, which causes a nuclear AKT activation even in the absence of insulin. A previous study showed that MYO1C (another isoform of myosin 1), in conjunction with RICTOR, was not involved in insulin-induced AKT activation in 3T3-L1 adipocytes (Hagan et al., 2008). Our study thus identified a function of MYO1B in inducing nuclear AKT activation.

The nuclear expression of AKT and its stimulated activation is controversial. Some studies demonstrate nuclear translocation of AKT upon stimulation (Nguyen et al., 2006, Pekarsky et al., 2000), whereas other studies show that AKT may be constantly localized and activated in the nucleus (Shiraishi et al., 2004, Wang and Brattain, 2006). Our results from both immunofluorescence staining and immunoblotting support the later finding. It has been several decades since nuclear AKT was demonstrated to be activated by growth factors, yet it is still unknown how nuclear AKT activation is regulated (Martelli et al., 2012). The results from the current study indicate that the rapid increase in nuclear AKT phosphorylation upon stimulation is unlikely to result from the translocation of the activated AKT from the cytoplasm into the nucleus. First, there is no change in the total nuclear AKT. Second, nuclear PI(3)P reflecting PI3K and PTEN activity (Downes et al., 2007) is enriched in the nuclear membrane and remarkably enhanced in parallel with the increase in cytoplasmic PI(3)P upon stimulation, suggesting the existence of the AKT activation mechanism in nucleus. In an attempt to elucidate the potential mechanism by which MYO1B regulates nuclear AKT activation, we investigated the role of PTEN, a well-known negative regulator of PI3K-AKT signal pathway, because nuclear PTEN has also been demonstrated for many years in melanoma cells and epithelial thyroid tumors (Gimm et al., 2000, Whiteman et al., 2002). Our results confirm the existence of nuclear PTEN in MEFs that are distributed throughout the nucleus. Both immunostaining and immunoblotting analysis of subcellular fractionation suggests that MYO1B regulates nuclear AKT activation by modulating subcellular distribution of PTEN. This conclusion is supported by several lines of experimental data. First, silencing MYO1B enhances nuclear but reduces cytoplasmic PTEN, which is well correlated with reduced nuclear PI(3)P reflecting PI3K and PTEN activity and AKT activation under the same experimental condition. Second, overexpression of MYO1B decreases nuclear PTEN with concomitant enhanced nuclear AKT activation. It is to be noted that silencing or overexpressing MYO1B does not affect cytoplasmic AKT activation despite the reduced or enhanced cytoplasmic PTEN, respectively. A possible explanation would be that the level of only non-functional MYO1B-bound form of PTEN, but not functional free PTEN in the cytoplasm, is affected by silencing or overexpressing MYO1B. The fact that insulin-induced activation of AKT2 isoform is most significantly suppressed by silencing MYO1B implies that this isoform most likely localizes in the nucleus. Third, MYO1B exerting its regulatory effect at the nuclear level of PTEN by binding to PTEN is dependent on the motor domain of MYO1B and a mutant in the motor domain deficient in its binding to PTEN fails to cause a change in subcellular distribution of PTEN. Fourth, most importantly, MYO1B overexpression-mediated suppression of cell apoptosis, which is associated with reduced nuclear PTEN level, is not observed when a mutant of PTEN harboring a nuclear localization signal is co-overexpressed with MYO1B. All these data provide firm evidence that MYO1B, by interacting with PTEN, prevents localization of PTEN in the nucleus, contributing to nuclear AKT activation and suppression of cell apoptosis. In addition to PTEN, protein phosphatase 2A (PP2A) and PH domain leucine-rich repeat protein phosphoserine (PHLPP) are well-characterized phosphatases that directly dephosphorylate and thereby inactivate AKT (Gao et al., 2005, Kuo et al., 2008, Rocher et al., 2007). Both PP2A and PHLPP are also present in the nucleus (Reyes et al., 2014, Turowski et al., 1995). It would be interesting to investigate further whether MYO1B modulates nuclear AKT activation also by the regulation of nuclear PP2A and/or PHLPP.

Despite the absence of a classical nuclear localization signal, PTEN has been reported to exist in the nucleus (Gimm et al., 2000, Perren et al., 2000, Sano et al., 1999). The fact that the loss of nuclear PTEN specifically was found in a variety of sporadic tumors suggests an important role of nuclear PTEN as a tumor suppressor. An impaired transport system of PTEN to the nucleus or some other means of differential compartmentalization has been proposed to account for impaired PTEN function as tumor suppressor (Perren et al., 2000). However, the mechanism(s) governing the nuclear-cytoplasmic partitioning of PTEN remains elusive. In this study, we demonstrate that MYO1B regulates the subcellular distribution of PTEN by binding to PTEN, which prevents the localization of PTEN in the nucleus. The interaction between PTEN and MYO1B are demonstrated by three different methods including co-immunoprecipitation, co-immunostaining, and PLA. However, these methods do not allow to conclude whether the interaction between PTEN and MYO1B is direct. More experiments will be required to address this aspect conclusively. As MYO1B is expressed in both nucleus and cytoplasm in immortalized MEFs, which subcellular MYO1B accounts for the regulation of subcellular distribution of PTEN remains to be investigated. However, the fact that MYO1B is mainly detected to co-localize with PTEN in the cytoplasm suggests that the binding of MYO1B to PTEN in the cytoplasm may prevent translocation of PTEN into nucleus. Further experiments need to be designed to confirm this conclusion. It is to be noted that the interaction of MYO1B and PTEN is not affected by short-time stimulation with insulin, indicating that the MYO1B-mediated regulation of nuclear-cytoplasmic partitioning of PTEN represents an insulin-independent mechanism.

Both nuclear AKT and PTEN exert a variety of cellular functions. Among these regulation of apoptosis is a common one shared by nuclear AKT and PTEN linking to tumorigenesis. In line with the antagonizing effect of PTEN on AKT activation, nuclear AKT has been shown to protect cells against apoptosis (Lee et al., 2008, Rubio et al., 2009), whereas nuclear PTEN promotes cell apoptosis and decreased nuclear PTEN has been correlated with progressing thyroid carcinoma and melanoma (Brenner et al., 2002, Chang et al., 2008, Depowski et al., 2001, Whiteman et al., 2002). In support of our conclusion that MYO1B prevents PTEN nucleus localization through interaction with PTEN dependent on its motor domain, the effects of silencing MYO1B or overexpressing MYO1B-WT or MYO1B-KA mutant on cell apoptosis fit well into their corresponding effect on nuclear PTEN level, i.e., MYO1B level is inversely associated with the nuclear PTEN level and cell apoptosis. These results are obtained in both immortalized MEFs and melanoma cells, demonstrating an anti-apoptotic role of MYO1B by preventing nuclear accumulation of PTEN. To this end, it is noteworthy that in our recently published study MYO1B is demonstrated to mediate ARG2-induced MTORC1-RPS6K1 leading to enhanced apoptosis in senescent vascular smooth muscle cells (Yu et al., 2018). The opposing effect of MYO1B on cell apoptosis in senescent vascular smooth muscle cells and melanoma cells suggest its dual role in the regulation of apoptosis through different mechanisms in different cells and circumstances. There are many other examples of molecules that can trigger both cell death and survival pathways. Particularly, oncogenes such as MYC, RAS, and E2F1, which deliver strong mitogenic signals, have also been reported to cause cell death (Knezevic et al., 2007, Kopnin et al., 2007, Tanaka et al., 2002). Regarding the role of MYO1B in cell apoptosis, it promotes apoptosis in senescent cells through ARG2-induced over-activation of MTORC1-RPS6K1 signaling (Yu et al., 2018), whereas it antagonizes apoptosis by preventing nuclear accumulation of PTEN in immortalized cells and tumor cells such as melanoma.

In summary, our current study uncovers a function for MYO1B as a regulatory mechanism of nuclear PTEN-AKT pathway linking to cell apoptosis. That is, by binding to PTEN dependent on its motor domain, MYO1B prevents the nuclear accumulation of PTEN leading to the activation of nuclear AKT and protection of cells from apoptosis. Targeting MYO1B may represent a therapeutic approach for cancer treatment such as melanoma.

### Limitations of the Study

In the present study, we identified MYO1B as a regulator of nuclear AKT activation. We demonstrate that MYO1B, by interacting with PTEN through the motor domain, prevents localization of PTEN in the nucleus, contributing to nuclear AKT activation and suppression of cell apoptosis. Although the interaction between MYO1B and PTEN has been demonstrated by three different techniques including co-immunoprecipitation, co-immunostaining, and PLA, these methods, however, do not allow to conclude whether the interaction between PTEN and MYO1B is direct. More experiments will be required to address this aspect conclusively.

## Methods

All methods can be found in the accompanying Transparent Methods supplemental file.

## Supplemental Information

Document S1. Transparent Methods and Figures S1–S5
