# Supplementary material for: Myosin 1b Regulates Nuclear AKT Activation by Preventing Localization of PTEN in the Nucleus
Source: iScience. 2019 Jul 11;19:39–53. doi: 10.1016/j.isci.2019.07.010 (PMC6660601; doi:10.1016/j.isci.2019.07.010)
Supplement: Document S1. Transparent Methods and Figures S1–S5 [file mmc1.pdf]

ISCI, Volume *19*

Supplemental Information

Myosin 1b Regulates Nuclear AKT Activation by Preventing Localization of PTEN in the Nucleus

Yi Yu, Yuyan Xiong, Diogo Ladeiras, Zhihong Yang, and Xiu-Fen Ming

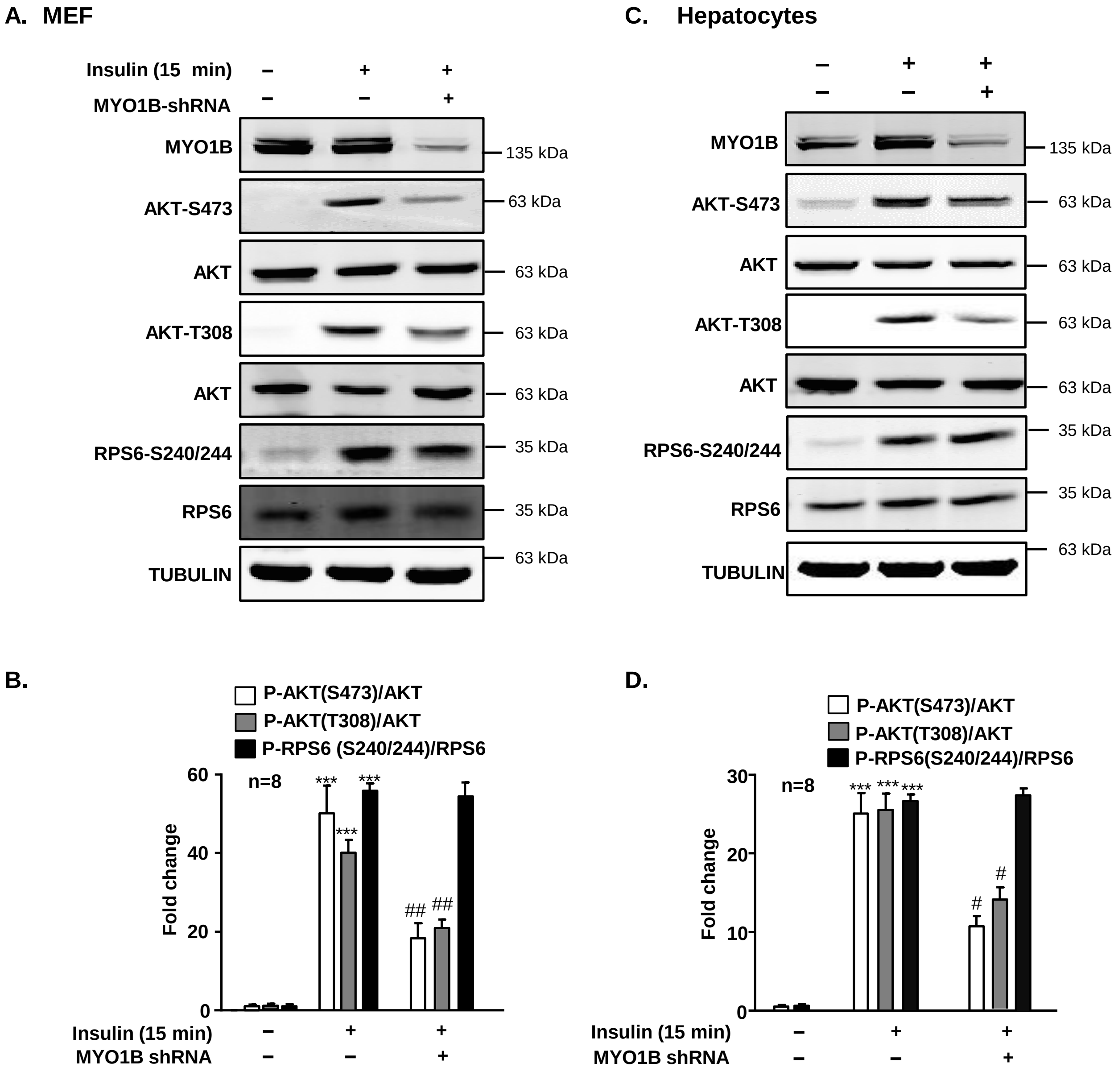

Figure S1. Silencing MYO1B inhibits short-term insulin-induced AKT-phosphorylation. Related to Figure 1. Immortalized MEF cells (A) and hepatocytes (C) were transduced with rAd/U6-LacZ- shRNA as control, rAd/U6-MYO1B-shRNA for silencing. After 3 days of transduction and 16 h of serum-starvation, the cells were treated with or without 100 nmol/L insulin for 15 min. Immunoblotting analysis of MYO1B, AKT phosphorylation, and S6 ribosomal protein phosphorylation are shown. TUBULIN served as loading control. Quantification of the signals in (A) and (C) is shown in the bar graphs in (B) and (D), respectively. All values are presented as mean ± SEM of the data from 8 independent sets of experiments. One-way ANOVA. ***P<0.001 vs control; #P<0.05, ##P<0.01 vs insulin in the absence of MYO1B-shRNA. rAd, recombinant adenovirus.

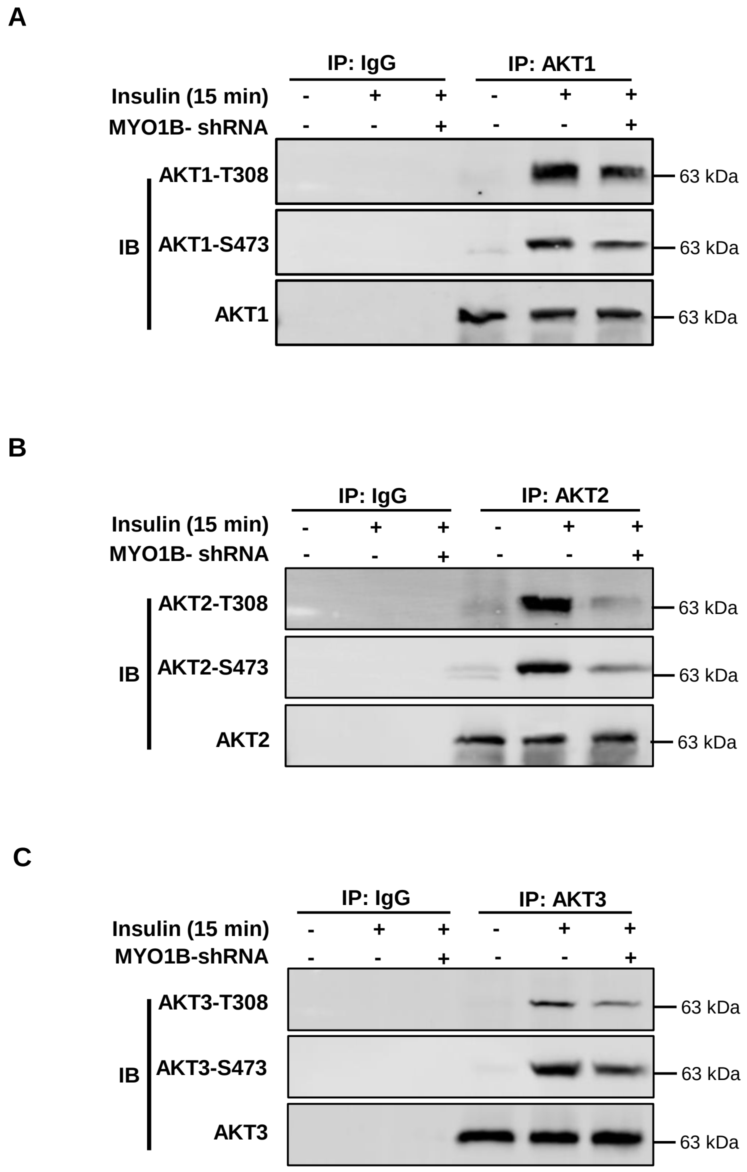

Figure S2. Silencing MYO1B inhibits insulin-induced AKT1/AKT2/AKT3-phosphorylation. Related to Figure 1. Immortalized MEF cells were transduced with rAd/U6-LacZ- shRNA as control, rAd/U6-MYO1B-shRNA for silencing. After 3 days of transduction and 16 h of serum-starvation, the cells were treated with or without 100 nmol/L insulin for 15 min. AKT1/AKT2/AKT3 was immunoprecipitated (IP) with an anti-AKT1/AKT2/AKT3 antibody. IgG was used as the negative control to verify the specificity of antibody. Immunoblotting analysis of AKT1/AKT2/AKT3-phosphorylation and total AKT1/AKT2/AKT3 were carried out with immunoprecipitates.

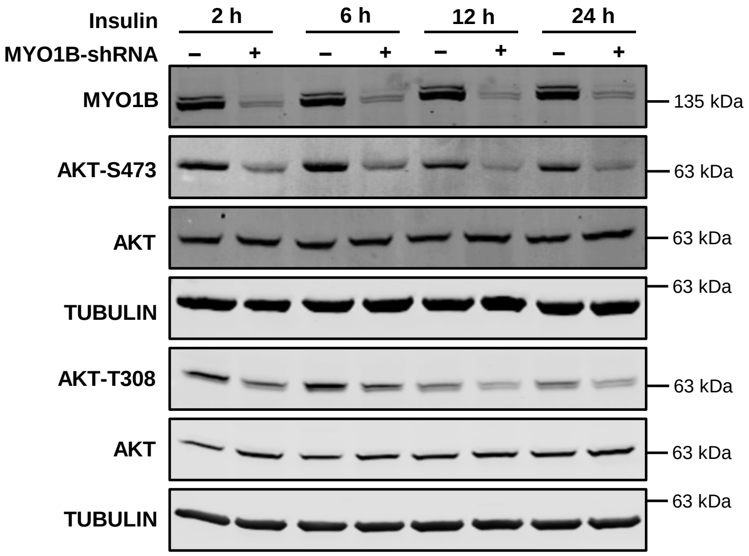

Figure S3. Silencing MYO1B inhibits long-term insulin-induced AKT activation. Related to Figure 1. Immortalized MEF cells were transduced with rAd/U6-LacZ-shRNA as control, rAd/U6-MYO1B-shRNA for silencing. After 3 days of transduction and 16 h of serum-starvation, the cells were treated with 100 nmo/L insulin for 2 h, 6 h, 12 h and 24 h. Immunoblotting analysis of MYO1B and AKT phosphorylation are shown.

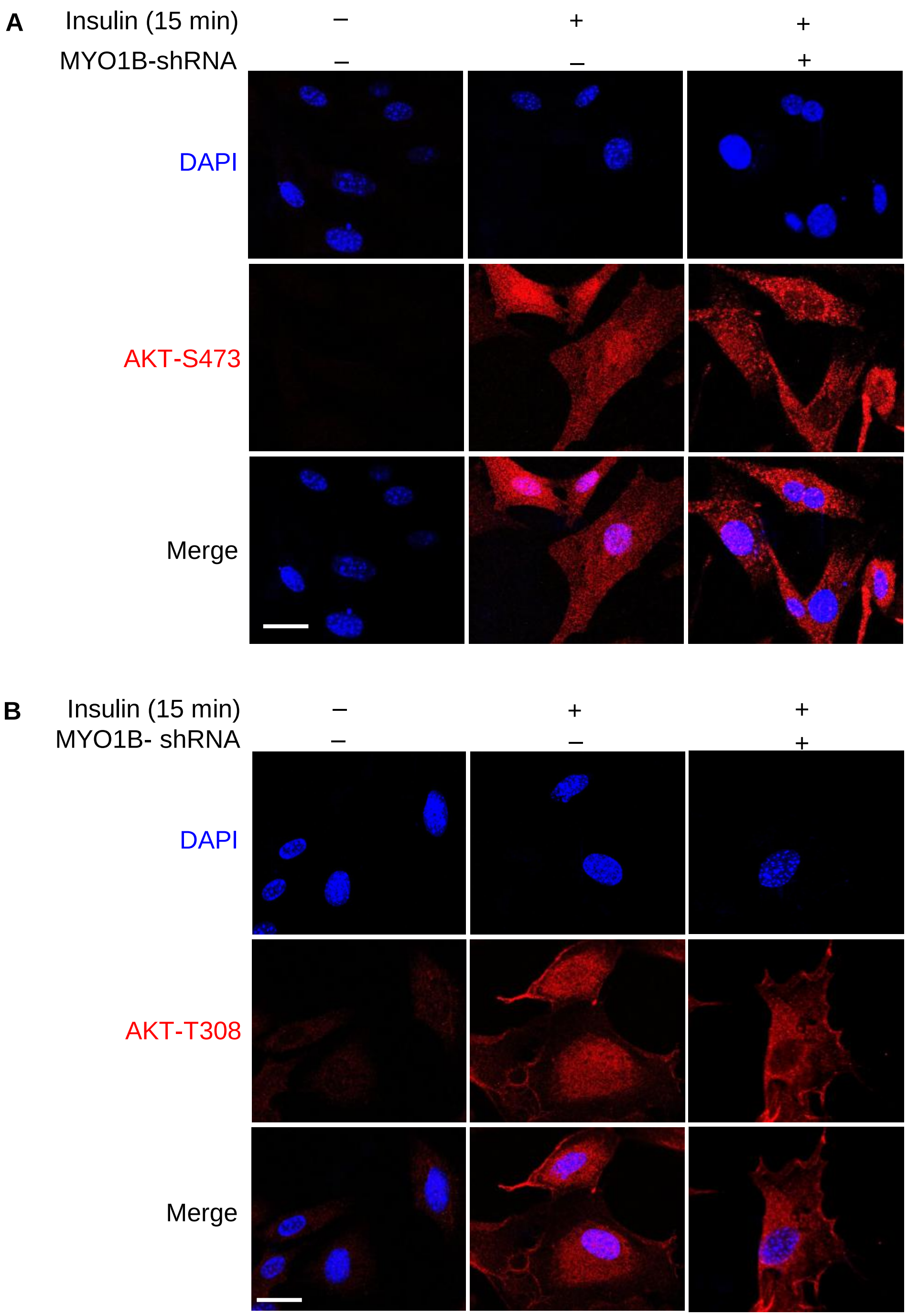

Figure S4. MYO1B is required for insulin-induced nuclear AKT activation. Related to Figure 1C and 1D. Immortalized MEF cells were transduced with rAd/U6-LacZ-shRNA as control, rAd/U6-MYO1B-shRNA for silencing MYO1B. After 3 days of transduction and 16 h of serum-starvation, the cells were treated with or without 100 nmol/L insulin for 15 min. **(A)** Immunofluorescence staining for AKT-S473 (red) and **(B)** AKT-T308 (red) were followed by counterstaining with DAPI (blue). The merged images are also shown. Scale bar = 25 µm.

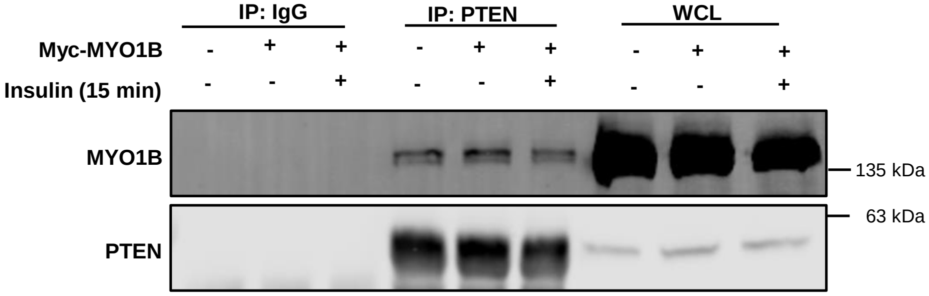

Figure S5. MYO1B interacts with PTEN independently of insulin. Related to Figure 4. Immortalized MEF cells were transduced with rAd/CMV-LacZ as control, rAd/CMV-MYO1B for overexpression. After 2 days of transduction and 16 h of serum-starvation, the cells were treated with or without 100 nmol/L insulin for 15 min. PTEN was immunoprecipitated (IP) with an anti-PTEN antibody. IgG was used as the negative control to verify the specificity of antibody. Immunoblotting analysis of MYO1B and PTEN were carried out with immunoprecipitates. WCL: whole cell lysate

TRANSPARENT METHODS

Materials

Reagents were purchased or obtained from the following sources: rabbit (sc-20151) and mouse (sc-393496) antibodies against Arg-II and mouse antibody against myosin 1b (MYO1B) (sc-393053) and PTEN (sc-7974) were from Santa Cruz Technology Inc (Dallas, USA); mouse antibodies against RPS6 (#2317s), AKT1 (#2967s), AKT2 (#5239s), and caspase 9 (#9508s), rabbit antibodies against AKT-Ser473 (#9271s), AKT-Thr308 (#13038s), phospho-RPS6-S235/236 (#2211s) and AKT3 (#14982s) were purchased from Cell Signaling (Danvers, USA); mouse antibody against AKT (610860) was from BD Transduction laboratories (New Jersey, USA); mouse antibody against PI(3)P (Z-P003) was from Echelon Biosciences Inc (Utah, USA); rabbit antibodies against LMNB1 (ab16048) and MYO1B (ab194356) were from Abcam (Cambridge, UK); Duolink® In Situ Detection Reagents Red (DUO92008) and mouse antibody against TUBULIUN (T5168) was from Sigma (St. Louis, Missouri, USA). IRDye 800-conjugated affinity purified goat anti-rabbit IgG F(c) was purchased from LI-COR Biosciences (Lincoln, Nebraska USA); goat anti-mouse IgG (H+L) secondary antibody Alexa Fluor® 680 conjugate, goat anti-mouse IgG (H+L) secondary antibody Alexa Fluor® 488 conjugate, goat anti-rabbit IgG (H+L) secondary Antibody Alexa Fluor® 488 conjugate and goat anti-rabbit IgG (H+L) secondary antibody Alexa Fluor® 594 conjugate were from Invitrogen/Thermo Fisher Scientific (Waltham, MA USA). Insulin-transferrin-selenite sodium and dexamethasone were from Sigma (St. Louis, Missouri, USA). All cell culture media and materials were purchased from Gibco/Thermo Fisher Scientific (Waltham, Massachusetts, USA).

Generation of recombinant adenovirus (rAd)

Generation of rAd expressing shRNA targeting mouse MYO1B driven by the U6 promoter (rAd/U6-mMYO1B shRNA) was carried out with the Gateway Technology. The targeting sequences are indicated in boldface below (only the sense strand is shown):

mMYO1B-shRNA:

5'- CACCGGAGCTCCTCTACAAGCTTAACGAATTAAGCTTGTAGAGGAGCTCC -3'

rAd/U6-LacZ shRNA served as control was generated (Yepuri et al., 2012).

Generation of rAd expressing PTEN fused with nuclear localization sequences (NLSs) of SV40 large T-Antigen driven by cytomegalovirus (CMV) promoter, rAd/CMV-HA-NLS-PTEN was also carried out with the Gateway Technology. The gene encoding HA-NLS-PTEN was amplified by polymerase chain reaction from the expression plasmid HA-PTEN (a gift from Jaewhan Song, Addgene plasmid # 78776) (Lee et al., 2015), by using following primers (the underline indicating the NLSs): Forward: 5'CTTAACCATGGTCGACCCAAAGAAAAAGAGAAAAGTAATGGCCTCCTACCCTTATGATGTG -3'; Reverse: 5'- ATCTCGAGTGCGGCCGCTCAGACTTTTGTAATTTGTGTATG-3'.

Generation of rAd expressing myc-MYO1B-WT and its mutants -R165A (deficient in its motor activity) and-K966A (deficient in its C-terminal PH domain) driven by CMV promoter (rAd/CMV-myc-MYO1B-WT, -R165A, -K966A) was also carried out with the Gateway technology. The expression plasmids

encoding myc-MYO1B-WT, -R165A and-K966A were kindly provided by Lynne M. Coluccio (Komaba and Coluccio, 2010).

Cell culture and adenoviral transduction

The immortalized MEFs were kindly provided by Dr. Jürgen A. Ripperger (Department of Biology, University of Fribourg) and the mouse melanoma B16F10 cell line by Dr. Carole Bourquin (Department of Immunopharmacology of Cancer, University of Geneva). MEF and B16F10 cells were cultured in high glucose-Dulbecco's Modified Eagle's Medium (D6429, Sigma) containing 10 % heat-inactivated fetal bovine serum (10500-064, Gibco) or 10% fetal calf serum respectively, and 1% penicillin-streptomycin. The alpha mouse liver 12 cell line (AML12) was purchased from ATCC (CRL-2254) and maintained in DMEM /Nutrient Mixture F-12 Ham supplemented with 10% HIFBS, insulin-transferrin-selenite sodium and dexamethasone (Liu et al., 2016). For insulin stimulation experiments, MEFs and AML12 were starved for overnight in low glucose DMEM (6046, sigma) containing 0.2% BSA and DMEM /Nutrient Mixture F-12 Ham containing 0.2% BSA, insulin-transferrin-selenite sodium and dexamethasone, respectively. Cells were transduced with the rAd at titers of ~200 multiplicities of infection and then cultured in complete medium for two days and then switched to serum-free medium overnight before experiments.

Immunoblotting

Cell extracts were prepared by lysing cells in lysis buffer (120 mM NaCl, 50 mM Tris [pH 8.0], 20 mM NaF, 1 mM benzamidine, 1 mM EDTA, 1 mM EGTA, 1 mM sodium pyrophosphate, 30 mM 4-nitrophenyl phosphate disodium salt hexahydrate, 1% NP-40, and 0.1 M phenylmethylsulfonyl fluoride [PMSF]). Next, 40-µg extracts were subjected to sodium dodecyl sulfate-polyacrylamide gel electrophoresis and electrophoretically transferred to an Immobilon-P membrane (Millipore), and the resultant membrane was incubated overnight with the corresponding primary antibody at 4°C with gentle agitation after being blocked with 5% skimmed milk (Yepuri et al., 2012). The blot was then further incubated with a corresponding anti-mouse (Alexa fluor 680 conjugated) or anti-rabbit (IRDye 800 conjugated) secondary antibody. Signals were visualized using Odyssey Infrared Imaging System (LI-COR Biosciences). Quantification of the signals was performed using NIH Image 1.62 software (U. S. National Institutes of Health).

Subcellular fractionation

Cells were transferred from 10 cm plates into 500 µL fractionation buffer (HEPES (pH 7.4) 20 mM, KCl 10 mM, MgCl2 2 mM, EDTA 1 mM, EGTA 1 mM, 1mM DTT, PI Cocktail (III)) by scraping followed by incubation on ice for 15 min. Cells were then lysed by passing cell suspension through a 27 gauge needle several times using a 1 mL syringe and kept on ice for 20 min. After centrifugation of the samples at 720 x g (3,000 rpm) for 5 min, the supernatant containing cytoplasm, membrane and mitochondria was transferred into a fresh tube and kept on ice. The pellet containing nuclei was washed with 500 µL fractionation buffer and dispersed with a pipette and passed through a 25 gauge needle 10 times followed by centrifugation again at 3,000 rpm for 10 min. The nuclei pellets were then

resuspended in TBS with 0.1% SDS and sonicated briefly to shear genomic DNA and to homogenize the lysate (3 sec on ice at a power setting of 2-continuous). For cytoplasm preparation, the supernatant containing cytoplasm, membrane and mitochondria was centrifuged at 8,000 rpm (10,000 x g) for 5 min. The supernatant containing cytoplasm and membrane was then transferred into a fresh tube and kept on ice or -80 ^{o}C until use.

In Situ Proximity Ligation Assay (PLA)

MEF cells cultured on coverslips were washed with PBS, and then incubated in ice cold 100% methanol for 10 minutes at –20 °C, rinse in PBS for 5 minutes, permeabilized in 0.3% Triton X-100 for 10 min, and blocked with Duolink Blocking Solution. After blocking, cells were incubated with combined primary antibodies (anti-MYO1B or Myc and anti-PTEN) overnight at 4 °C. Cells were then incubated with the PLUS and MINUS PLA probes diluted 1:5 in the Duolink Antibody Diluent in a pre-heated humidified chamber for 1 h at 37 °C. Subsequent ligation, amplification, and detection were performed according to manufacturer's instruction. Fluorescence images were acquired using a Leica TCS SP5 confocal laser microscope and the signals of PLA were quantified with NIH Image 1.62 software (U. S. National Institutes of Health).

Immunoprecipitations

MEF cells growing in 10 cm dishes were rinsed once with cold PBS and lysed on ice for 20 min in 1 ml of ice-cold lysis buffer (40 mM HEPES [pH 7.5], 120 mM NaCl, 1 mM EDTA, 10 mM pyrophosphate, 10 mM glycerophosphate, 50 mM NaF, and EDTA-free protease and phosphatase inhibitors) containing 0.3% CHAPS. After centrifugation at 13,000 x g for 10 min, the protein concertation of cleared supernatant was measured by the DC™ Protein Assay (5000112, Bio-Rad). 5 μg of the indicated antibodies were added to the 800 μg protein supernatant and incubated with rotation overnight at 4 °C. 20 μL of Protein A/G PLUS-Agarose (sc-2003, Santa Cruz Biotechnology) was then added and the incubation continued for 2 h at room temperature. Pulled-down immunoprecipitates were then washed three times with lysis buffer. Samples were resolved by SDS-PAGE and proteins transferred to PVDF and visualized by immunoblotting.

Immunofluorescence staining

Cells cultured on glass coverslips were fixed with 4% paraformaldehyde for 15 min at room temperature and then permeabilized with 0.2% Triton X-100, and blocked with 1% BSA in PBS for 60 mins. Coverslips were incubated with corresponding primary antibody overnight at 4°C, followed by incubation with Alexa Fluor-labeled secondary antibodies for 2 h at room temperature, and mounted. Images were acquired through 40×objectives with Leica TCS SP5 confocal laser microscope. Representative images taken at the same exposure and magnification are shown in all figures.

Detection of apoptotic cells

Apoptosis of transduced MEFs was detected with Annexin-V-FLUOS Staining Kit (Roche Applied Science, #1988549) according to the manufacturer’s instructions. Quantification was presented by the ratio of apoptotic cells/total cells.

Viable cell count

Cells were seeded in a 6 well-plate at a density of 8 x 10^4 cells/well and allowed to attach overnight. After 3 days post-transduction, cells were collected by trypsinization and stained with 0.4% Trypan blue solution (T8154, Sigma). Unstained viable cells were counted using the TC20™ Automated Cell Counter from Bio-Rad Laboratories (California, USA) according to the manufacturer’s instruction. Each independent experiment was performed in triplicate.

Statistics

Data are given as mean ± SEM. In all experiments, n indicates the number of individual experiments. Statistical analysis was performed with unpaired Student t test or ANOVA with Dunnett or Bonferroni post-test. Differences in mean values were considered significant at $p < 0.05$.

SUPPLEMENTAL REFERENCES

Komaba, S., and Coluccio, L.M. (2010). Localization of myosin 1b to actin protrusions requires phosphoinositide binding. J. Biol. Chem. 285, 27686-27693.

Lee, M.S., Jeong, M.H., Lee, H.W., Han, H.J., Ko, A., Hewitt, S.M., Kim, J.H., Chun, K.H., Chung, J.Y., Lee, C., et al. (2015). PI3K/AKT activation induces PTEN ubiquitination and destabilization accelerating tumourigenesis. Nature Communications 6.

Liu, C., Rajapakse, A.G., Riedo, E., Fellay, B., Bernhard, M.C., Montani, J.P., Yang, Z., and Ming, X.F. (2016). Targeting arginase-II protects mice from high-fat-diet-induced hepatic steatosis through suppression of macrophage inflammation. Sci. Rep. 6, 20405.

Yepuri, G., Velagapudi, S., Xiong, Y.Y., Rajapakse, A.G., Montani, J.P., Ming, X.F., and Yang, Z.H. (2012). Positive crosstalk between arginase-II and RPS6K1 in vascular endothelial inflammation and aging. Aging Cell 11, 1005-1016.
